# Functional Connectivity Analysis on Resting-State Electroencephalography Signals Following Chiropractic Spinal Manipulation in Stroke Patients

**DOI:** 10.3390/brainsci10090644

**Published:** 2020-09-17

**Authors:** Toby Steven Waterstone, Imran Khan Niazi, Muhammad Samran Navid, Imran Amjad, Muhammad Shafique, Kelly Holt, Heidi Haavik, Afshin Samani

**Affiliations:** 1 Department of Health Science and Technology, Aalborg University, 9000 Aalborg, Denmark; tobywaterstone@gmail.com (T.S.W.); imran.niazi@nzchiro.co.nz (I.K.N.); m.navid@rn.dk (M.S.N.); 2 Centre for Chiropractic Research, New Zealand College of Chiropractic, Auckland 1060, New Zealand; imran.amjad@nzchiro.co.nz (I.A.); kelly.holt@nzchiro.co.nz (K.H.); Heidi.Haavik@nzchiro.co.nz (H.H.); 3 Faculty of Health & Environmental Sciences, Health & Rehabilitation Research Institute, AUT University, Auckland 1010, New Zealand; 4 Faculty of Rehabilitation and Allied Sciences & Faculty of Engineering and Applied Sciences, Riphah International University, Islamabad 44000, Pakistan; muhammad.shafique@riphah.edu.pk

**Keywords:** chiropractic, stroke, resting-state electroencephalography, functional connectivity, spinal manipulation

## Abstract

Stroke impairments often present as cognitive and motor deficits, leading to a decline in quality of life. Recovery strategy and mechanisms, such as neuroplasticity, are important factors, as these can help improve the effectiveness of rehabilitation. The present study investigated chiropractic spinal manipulation (SM) and its effects on resting-state functional connectivity in 24 subacute to chronic stroke patients monitored by electroencephalography (EEG). Functional connectivity of both linear and non-linear coupling was estimated by coherence and phase lag index (PLI), respectively. Non-parametric cluster-based permutation tests were used to assess the statistical significance of the changes in functional connectivity following SM. Results showed a significant increase in functional connectivity from the PLI metric in the alpha band within the default mode network (DMN). The functional connectivity between the posterior cingulate cortex and parahippocampal regions increased following SM, *t* (23) = 10.45, *p* = 0.005. No significant changes occurred following the sham control procedure. These findings suggest that SM may alter functional connectivity in the brain of stroke patients and highlights the potential of EEG for monitoring neuroplastic changes following SM. Furthermore, the altered connectivity was observed between areas which may be affected by factors such as decreased pain perception, episodic memory, navigation, and space representation in the brain. However, these factors were not directly monitored in this study. Therefore, further research is needed to elucidate the underlying mechanisms and clinical significance of the observed changes.

## 1. Introduction

Stroke is a common problem affecting people worldwide and it is the number one cause of chronic disability [1]. The disabilities that stroke survivors experience are often chronic and can present as both impaired cognitive and motor function [2]. Rehabilitation of stroke patients, especially in the early stages after the acute phase, is therefore of great importance, as mechanisms like neuroplasticity play a major role in the recovery, as the brain reorganizes and adapts to the lesion that the stroke has caused [3,4]. Rehabilitation methods applied during the early stages of recovery have been shown to improve the chances of a successful recovery [4]. 

During the past decades, a growing body of research has been focused on chiropractic spinal manipulation (SM) and its effects on the central nervous system (CNS) [5,6,7,8,9,10,11,12,13,14]. Research suggests that SM alters mechanoreceptive input from the spine and that this in turn alters the way in which the brain processes, interprets, and integrates other interoceptive and exteroceptive information [5]. Previous studies have investigated the effect of SM on the function of the nervous system at different levels, for example motor output, sensory processing, functional performance, and sensorimotor integration [5,15,16]. These studies have contributed to the hypothesized model, described by Haavik and Murphy [5], which proposes potential mechanisms to explain how SM may alter bodily and CNS function [5].

A recent study by Holt et al. 2019 [16] demonstrated that SM-altered neural activity in chronic stroke patients. This study reported an increase in cortical drive, measured by an increase in V-wave amplitude, along with increased force production of planter flexor muscles following SM. The mechanisms by which spinal manipulation could result in this increased cortical drive and greater maximum voluntary force production is not yet well understood. No previous study has explored brain connectivity changes in a stroke population following spinal manipulation.

Previously, the effects of SM within the brain has mainly been investigated in subjects suffering from pain. Using proton magnetic resonance spectroscopy in 25 patients with non-specific chronic low back pain, SM resulted not only in a decrease in pain, but also a significant increase in N-acetyl aspartate in the thalamus, insula, and dorsolateral prefrontal cortex regions, as well as a significant increase in choline in the thalamus, insula, and somatosensory cortex regions [17].

Using functional magnetic resonance imaging (FMRI), Gay et al. 2014 found changes in functional connectivity within the pain processing network (PNN) along with a decrease in pain perception after SM therapy in subjects with experimentally induced low back pain. In this group, the SM intervention led to increased functional connectivity between the left anterior insular cortex and left posterior cingulate cortex, the left posterior insular cortex and left periaqueductal gray, the right anterior insular cortex and right somatosensory cortex, the right anterior insular cortex and left posterior cingulate cortex, and a decrease in functional connectivity between the left somatosensory cortex and the right posterior insular cortex [18]. It is possible these intracortical changes are not only responsible for the decrease in pain, but may also reflect sensorimotor processing and integration changes that result in the increased cortical drive and maximum voluntary contraction found by other authors following SM [7,8,16]. 

Investigations of functional connectivity have become more common over recent years, as they may reveal the interconnection between various nuclei in the brain, thereby shedding light on neural pathways in the brain and how they function [19,20]. In particular, the default mode network (DMN) activity and functional connectivity between brain regions in this network have drawn special attention when examining cognitive dysfunction in psychiatric and neurologic brain disorders [21,22]. Several studies have found a decreased functional connectivity within the DMN in stroke patients compared to healthy subjects [18,23,24,25]. In a healthy brain, the DMN is one of the essential networks that is activated during rest and deactivated during task related work. Disruptions during rest within this network are linked with neurologic diseases such as stroke [21]. It is also highly likely that pain influences the functional connectivity in this brain network [24]. The DMN is of special interest when studying resting-state data, as specific brain regions related to this network have been shown to be more active during rest in healthy subjects [18,20].

Functional connectivity is believed to be expressed as both linear and non-linear processes within the brain and different measures of functional connectivity have been applied to electroencephalography (EEG) signals in the literature [26,27]. One classic approach, coherence, which measures linear associations between signals, has been widely used to measure EEG functional connectivity [26,27,28]. Other approaches, such as phase lag index (PLI), measure non-linear relationships between signals. PLI has been recommended for measuring functional connectivity, because it is less susceptible to volume conduction effects [29].

Even though functional connectivity can be analyzed from fMRI measurements, EEG is a superior method to use to study functional connectivity when it comes to temporal resolution, accessibility, and examination costs [27,30]. Using resting EEG to study functional connectivity has become increasingly popular over recent years [18,31,32]. Several studies have already utilized resting-state data in order to study functional connectivity in stroke survivors [31,32]. In order to see whether neurophysiological changes are present within the cortex following a single session of SM and to shed light on the potential of EEG as a method for monitoring these changes, the aim of this study is therefore to explore the effects of SM in subacute to chronic stroke patients by performing functional connectivity analysis on EEG signals at rest. This current study should be viewed as an exploratory study, where the feasibility of investigating functional connectivity based on EEG recording was assessed to examine whether this method can be used to investigate changes at the cortical level after an SM intervention in people with stroke. This will help shed light on the potential for EEG to measure the effects of SM on a neuroplastic level.

Based on prior SM studies and Gay et al. 2014 [18], it is hypothesized that functional connectivity will increase within the cortex of subacute to chronic stroke patients following SM.

## 2. Material and Methods

The study used a randomized controlled crossover design and was conducted at Railway General Hospital in Rawalpindi, Pakistan. The Riphah International University Research Ethics Committee, Pakistan, approved the study (ref # Riphah/RCRS/REC/000118). The study was also approved by the New Zealand College of Chiropractic Research Committee. The study was conducted in accordance with the Declaration of Helsinki.

### 2.1. Subjects

Twenty-four subacute to chronic stroke patients were recruited to participate in the experiment—all male, mean age 51.9 ± 11.4. The patients had suffered from a stroke between 3–60 months prior to participating in this study (mean 18.2 ± 14.4 months). The location of the stroke varied between patients. Thirteen subjects had suffered from a right hemisphere stroke and eleven from a left hemisphere stroke. Individual patient characteristics are listed in Table 1. 

### 2.2. Experimental Protocol and Equipment

**Figure 1:**
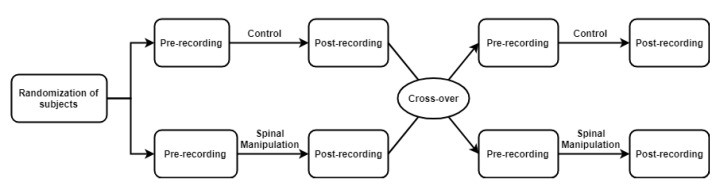
Illustration of the crossover study design and recording allocation.

The experiment was carried out as a randomized crossover study, where each participant took part in a control and an SM session in random order, separated by at least 24 h. An overview of the crossover study design is illustrated in Figure 1.

Resting-state EEG signals were recorded before and after the SM and the control procedures. Unipolar EEG signals were recorded using a 72 channel Refa TMSi EXG amplifier (TMSi, Enschede, The Netherlands), connected to a 64 electrode EEG cap placed on the scalp of the subjects according to the extended 10–20 system [33]. The setup was connected to ground through an electrode at location Fz. The recording setup was powered by a battery source in order to minimize power line noise. During the EEG acquisition, the subjects were positioned sitting comfortably in a chair, asked to sit still with eyes open and relaxed, keeping their gaze on a cross located on the wall 1.5 m away. During the two to three minutes of resting-state EEG, subjects were asked to keep movement and eye blinks to a minimum. Sampling frequency was set to 2048 Hz. Subjects returned for a second session at least 24 h after their first assessment to repeat the experiment with the alternate intervention to which they were blinded. At the end of the second session, the subjects were asked if they perceived that they had undergone active treatment in each session (‘yes’ or ‘no’).

### 2.3. Intervention

Two interventions were performed during the study, these involved a spinal manipulation session and a control session. These interventions have been used in similar studies [7,13,14,16,34]. The SM intervention and control procedures each took approximately 10–15 min, thus the time between pre- and post-recordings for each intervention was approximately 15 min. 

#### 2.3.1. Chiropractic Spinal Manipulation Session (SM)

The subject’s spine and pelvic joints were assessed for vertebral subluxations (also known as spinal dysfunction) using standard clinical indicators routinely used in chiropractic practice [35,36]. These indicators included restricted intersegmental range of motion, soft tissue tension, leg length inequality, joint tenderness to palpation, and/or other abnormal joint play. The SM intervention consisted of high velocity, low amplitude chiropractic adjustments that were directed at the level of the vertebral subluxations and were delivered by hand, or by using a spring-loaded, hand-held mechanical instrument. Multiple segments were adjusted during the intervention session if deemed to be clinically warranted.

#### 2.3.2. Control Session 

During the control session the subjects were examined using the same procedures used in the SM session. They were then moved into position to be adjusted, but no thrust was applied to the spine or pelvis. This procedure was performed to account for changes, which could happen due to muscular, cutaneous, or vestibular effects that may be related to the movement and touch used during the SM session. This procedure was done between pre- and post-recordings by the same chiropractor, similar to the study done by Holt et al., 2019 [16].

### 2.4. Data Analysis

**Figure 2:**
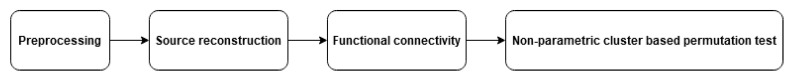
Pipeline illustrating the steps performed during the data analysis.

Offline data analysis was conducted in MATLAB R2018a. A pipeline of the methodological approach for the data analysis is outlined in Figure 2.

#### 2.4.1. Preprocessing

EEGLAB version 14.1.2 [37] was used for the entire preprocessing procedure. Initially a digital bandpass filter of zero phase finite impulse response (FIR) was applied to the signals using a hamming window. The filter was defined with the bandwidth 1–45 Hz of order specified automatically in EEGLAB by an heuristic process, which determined the order based on the high edge frequency and sampling rate [38,39].

During the preprocessing, re-referencing was done to achieve common average reference, excluding the two mastoid references, leaving 62 electrodes for further analysis. Before artifact removal, bad channels were marked using EEGLAB plug-in clean raw data, which marked the data by searching for flat line, noisy signals with excessively large amplitudes and poorly correlated neighboring channels. The channels marked as bad were manually inspected, validated, and excluded if the channels were considered bad [40]. On average, two channels were removed from the datasets, maximum of four across all trails. Artifact subspace reconstruction (ASR) were used to mark artifacts in the data, with a cutoff parameter threshold of 200, as recommended by Chang et al. 2018 [40]. The signals determined by the ASR algorithm was removed after visual inspection, in order to validate the marked artifacts. After rejection of bad channels and artifact removal, channel count would be unequal for individual subjects, which would be the source of error in subsequent processing if not handled properly [41]. Therefore, the removed channels were reconstructed using spherical interpolation, where information from neighboring channels was used for reconstruction after artifact removal [42]. Lastly, independent component analysis (ICA) was used to remove noise from eye blink, eye movement, and muscle activity [43]. Initially, the EEG signals were down-sampled to a sampling rate of 512 Hz, in order to optimize computation efficiency. The ICA components were semi-automatically marked as bad or good components using the multiple artifact rejection algorithm (MARA) [43]. MARA is an opensource EEGLAB plugin, based on a supervised machine learning algorithm, which automatically labels the independent components for artifact rejection. The components were visually inspected and verified before removal [43,44]. For further analysis of functional connectivity, 60 s of artifact-free signal were selected from all datasets [45].

#### 2.4.2. Source Reconstruction

EEG signals at the sensor level do not seem appropriate for analysis of functional connectivity due to volume conduction [46], as the sensor signals are an expression of a complex mixture of overlapping signals from a number of brain regions [47]. Therefore, EEG sources reconstruction was performed using Brainstorm version May 2019 in MATLAB R2018a [27,47,48]. EEG source reconstruction is a strategy to solve the problem of volume conduction, while also enhancing the spatial resolution of the data. The cortical activity at the sources was modeled as electrical dipoles, and the EEG signal at the sensor level was assumed to be a mixture of multiple source signals [27]. EEG source reconstruction consisted of two main problems. Forward modeling and inverse modeling, which were dependent on each other for correct source reconstruction. Forward modeling involved the calculations and modeling of the human head, including scalp, skull and cortex, and sensor array electromagnetic properties, while the inverse problem used information from the forward modeling problem in order to identify most likely locations and strengths of cortical activity [27,48].

**Figure 3:**
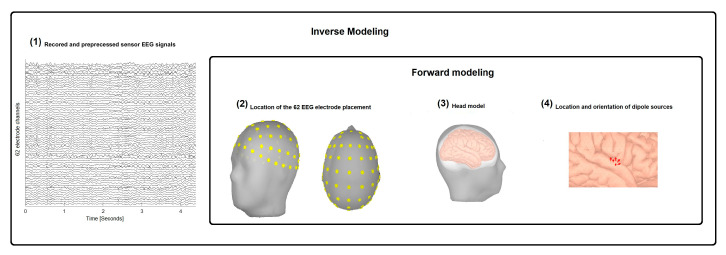
Illustration of the four essential pieces of information needed to do EEG source reconstruction. (**1**) Illustrating the EEG sensor time series, (**2**) the electrode placement, (**3**) the head model, (**4**) dipoles location and orientation.

For the EEG source reconstruction, four essential pieces of information were needed. (1) The sensor EEG signals, (2) information about the electrode placement on the head in 3D space, (3) a head model containing information about electrical and geometric characteristics of the head, and (4) a model that provides information about the location and orientation of dipole sources, which are being estimated [47]. Figure 3 illustrates the four pieces of information needed in order to achieve EEG source reconstruction.

Post-synaptic potentials generated in cortical pyramidal neurons of the cerebral cortex were assumed to be orientated approximately normal to the cortex; the mass effect of these neurons were computationally modeled [27].

#### 2.4.3. Forward Modeling

For the forward modeling, the location and orientation of current dipoles needed to be defined in order to define the EEG sensors in relation to the cortical source. This was done to define the location and orientation of source dipoles on a voxel grid space, resembling an approximation of the cortical space [48,49]. The set of dipoles were oriented perpendicular to the cortex, which was modeled by 15,000 vertices in a default generic head model from Brainstorm May 2019 using a symmetric boundary element method (Open MEEG BEM) for all subjects [48]. The head model used a three-layer compartment, i.e., scalp, skull, and brain. The tissues’ conductivities were defined based on the previous study by Lin and Scott 2012 [49], i.e., scalp = 1, Skull = 0.0125, and Brain = 1. The forward model was calculated after defining and locating the 62 electrode locations on the scalp. Locations of the electrodes were defined according to the 10–20 electrode placement system, using the Colin27 generic ASA 10–20 locations [33,50,51].

#### 2.4.4. Inverse Modeling

In the inverse problem, the activity from the 62 EEG sensors were estimated from the defined dipoles in the forward model, hence the inverse problem was defined from the forward problem.

The inverse problem is an ill-posted underdetermined problem, as the number of estimated sources are greater than the number of electrodes from the EEG sensor recordings. In order to achieve a solution for this problem, the method of minimum-norm solution was utilized by applying a linear kernel in the form of a matrix that multiplied the spatial data at each point in time. This method estimated cortical current source densities, which fitted approximately to the data of the forward model, by minimizing overall power of the activity from the estimated sources, with an identity matrix as noise covariance matrix [27,48]. To counteract the tendency of minimum norm estimate to locate the sources in superficial regions of the cortex, standardized low resolution brain electromagnetic tomography (sLORETA) was applied to normalize the current density maps of the source dipoles [52].

With sources constrained to be perpendicular to the cortex, the sources were represented as normalized current densities perpendicular to the cortex. The number of reconstructed sources were of very high resolution and would be inefficient to be used for the calculation of functional connectivity. Therefore, the sources were clustered based on the Desikan Killiany atlas with regions of interest (ROIs) on the cortex surface. This atlas defined 68 ROIs on the cortex surface. The sources were clustered as the average time series within the pre-defined ROIs, making up a [ROIs x time] matrix [47,48]. Before averaging, the sign of the dipoles with opposite direction were flipped, in order to avoid cancellation of activity because of opposite direction of dipole sources [47,53].

#### 2.4.5. Functional Connectivity Analysis

Prior to the calculation of functional connectivity, the DMN was derived from the sources only considering brain regions within this network, using the same brain regions as in the study from Kabbara et al. [38]. The brain regions considered within the DMN are listed in Table 2. 

**Figure 4:**
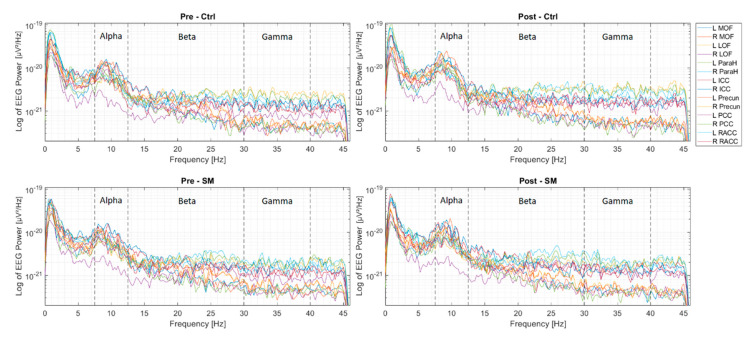
The four plots illustrate the power spectral density (PSD) estimations for the 14 regions within the default mode network (DMN) averaged across subjects, pre control session, plot (1); post control session, plot (2); pre SM session, plot (3), and post SM session, plot (4). Alpha, beta, and gamma frequency bands were specified in each plot. Left medial orbitofrontal (L MOF), right medial orbitofrontal (R MOF), left lateral orbitofrontal (L LOF), right lateral orbitofrontal (R LOF), left parahippocampal (L ParaH), right parahippocampal (R ParaH), left isthmus cingulate cortex (L ICC), right isthmus cingulate cortex (R ICC), left precuneus (L Precun), right precuneus (R Precun), left posterior cingulate Cortex (L PCC), right posterior cingulate cortex (R PCC), left rostral anterior cingulate cortex (L RACC), and right rostral anterior cingulate cortex (R RACC).

A power spectral density (PSD) analysis was performed to inspect the source reconstructed signals within the DMN in the frequency domain. To estimate the PSD for the frequency content of interest, the Welch method was used by estimating the PSD for every two seconds (2048 samples) of the signal applied with a Hanning window and 50% overlap between window segments [54,55]. The average PSD across these windows was then calculated, which made up the final PSD estimate. Figure 4 shows the PSD averaged across the two sessions for each condition.

• Coherence Analysis

Coherence measures similarities between two signals to reflect their connectivity and represents the linear correlation between these [27]. Coherence is bounded between zero and one, where zero indicates no coupling between signals and one reflects a linear relationship between signals. 

Magnitude squared coherence was calculated between each source in a pair for the alpha, beta, and gamma bands using Equation (1), where the Welch method was used to calculate the PSD [54].

(1) $$Coh_{x,y}\left(f\right)=\frac{{\left|PSD_{x,y}\left(f\right)\right|}^{2}}{PSD_{x}\left(f\right).PSD_{y}\left(f\right)},$$

In the equation, $Coh_{x,\;y}\left(f\right)$ was the magnitude squared coherence calculated between signals *x* and y at a specific frequency f, from the estimated PSD at the specified frequency for each signal, $PSD_{x}\left(f\right)$ and $PSD_{y}\left(f\right)$. $PSD_{x,y}\left(f\right)$ is the cross-signal PSD [54]. Coherence was calculated using a Hanning window of two seconds (2048 samples) and 50% overlapping windows in order to keep consistency with the PSD estimations. After the computation of coherence between every source pair, the coherence computed for each two second epochs were averaged across the 30 epochs to give one value representing the coherence value per source pair. The coherence data were stored in an adjacency matrix of dimension 14 × 14 with zeros along the diagonal, giving a symmetric square matrix, corresponding to the number of sources.

• Phase Lag Index

PLI quantifies connectivity as a measure of asymmetry calculated from the phase difference distribution between two signals *φ_x_*(*t*) and *φ_y_*(*t*) and measures non-linear coupling between signals, yielding phase differences being symmetrically distributed around zero in case of common source phase synchronization and therefore accounts for the effects of volume conduction (14). In order to estimate the PLI, the average phase difference was calculated using Equation (2), where the phase information *φ* of the signal was derived based on the phase of the ratio of the signals Hilbert transform and the signal itself [27]. The sign determined if the phase difference is positive, negative, or zero value, and *N* was the total number of samples contained within the windows, which the PLI was calculated over [27].

(2) $$PLI_{x,y}=\left|\frac{1}{N}\sum_{t=1}^{N}sign\left[sin\left(\varphi_{x}\left(t\right)-\varphi_{y}\left(t\right)\right)\right]\right|,$$

Before the computation of PLI, the data were divided into narrow band signals using a 4th order Butterworth filter in order to acquire the three frequency bands, alpha, beta, and gamma. According to the study by Newson and Thiagarajan 2019 [56], the frequency bands of EEG sources of the brain were defined from the reported ranges, alpha (7.5–12.5 Hz), beta (12.5–30 Hz), and gamma (30–40 Hz). The computation of PLI was done between every reconstructed EEG source signal in a pair. The result of the computed PLI also lies in the interval from zero to one, where zero indicates no coupling and one indicates perfect coupling between signals. PLI was calculated over four-second non-overlapping epochs, equivalent to 2048 samples, making up 15 epochs per source pair [57]. After the computation of PLI between every source pair, the PLI computed for each four second epoch was averaged across the 15 epochs to give one value representing the PLI value per source pair. The PLI data were stored in an adjacency matrix of dimension 14 × 14 with zeros along the diagonal, giving a symmetric square matrix, corresponding to the number of sources.

• Non-Parametric Cluster-Based Permutation Test

In order to handle the multiple comparison problem and control the familywise error, a non-parametric cluster-based permutation test was performed [58]. The calculated PLI values within the adjacency matrices was compared using a within-study design across control (pre to post) and SM (pre to post). The non-parametric cluster-based permutation test was performed utilizing the toolbox Field Trip (Donders Institute, Brain, Cognition and Behavior, Nijmegen in the Netherlands) by empirically estimating the null-distribution of a test statistic derived from a permutation of pooled PLI values across conditions. The test statistics were also computed to compare the conditions by means of a *t*-value extracted from a t-test comparing two conditions. A permutation *p*-value was obtained by calculating the fraction of the test statistics under null-hypothesis, which were larger than the test statistics derived under the experimental condition. 

The distribution of the test statistics under the null-hypothesis was obtained using Monte Carlo sampling with 5000 permutations at a significance level of 0.05 [58,59]. For each of the permutations, the t-values, which exceeded the critical t-value corresponding to the alpha-level of 0.05, were clustered in sets depending on their spatial adjacency. The calculated t-values within a cluster were summed, but since we adopted a two-sided test, we separated positive and negative t-values within a cluster. The t-values within a cluster, consisting of $b_{n}$ number of brain regions, were summed (Equation (3)) to compose the cluster level statistics $t_{m}$.

(3) $$t_{m}=\sum_{i=1}^{b_{n}}t_{i},$$

The largest cluster level statistic was utilized as the critical t-value at the cluster level. Similar cluster level statistics were calculated for the experimental condition and compared with the critical t-value at the cluster level for each of the permutations. For each of the clusters in the experimental conditions, a p-value was calculated as the fraction of the number of permutations whose critical t-value at the cluster level was larger than the obtained t-value of the experimental cluster. Thus, positive clusters (+clusters) indicated an increase in functional connectivity, while negative clusters (−clusters) indicated a decrease. In case of significant results, the effect size was calculated using the equation of Cohen’s effect size, Equation (4).

(4) $$d=\frac{M_{1}-M_{2}}{s_{1}},$$

where *d* indicated the effect size, *M* the mean of the value for the condition (pre or post) or group (Control or SM) and *s*_1_ the standard deviation of M1 [60]. Prior to the cluster-based permutation test, the coherence and PLI adjacency matrices were truncated to include only 20% of the strongest connections independently for each subject, eliciting network structures of nonrandom structure, as the strongest connectivity counts have been shown to reflect greater information of the underlying network architecture [61,62]. 

The cluster-based permutation test was then computed to look for differences within the adjacency matrices [22,38,63]. The results are visualized within the cortex using EEGNET version 1 [64]; where statistically significant results are illustrated as red dots representing the brain region and connecting lines representing statistical dependencies from the functional connectivity metrics between brain regions [64,65].

## 3. Results

Data analysis was performed on all 24 subjects, who had a minimum of 60 s of clean resting-state EEG in every session after cleaning of the data. From the questions to evaluate the success of subject blinding, out of the 24 subjects, three perceived that one of the sessions was not an active session, and one of these was correct in the identification of the order of the interventions he received (SM or control).

For functional connectivity calculated from PLI, an increased functional connectivity within the DMN was observed in the SM session in a number of brain regions, forming clusters in the non-parametric cluster-based permutation test, from pre- to post-recordings, whereas no significant changes were observed in the control session. No significant results in functional connectivity were found from the coherence analysis. The results are illustrated in Table 3.

### SM—Alpha Band

**Figure 5:**
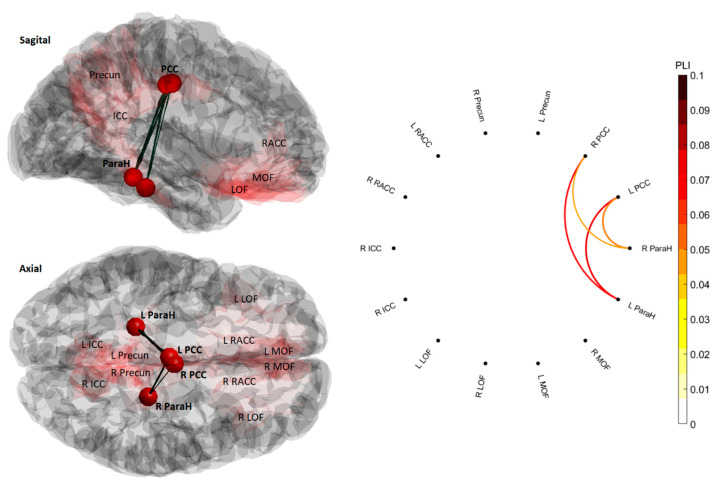
Illustration of the alpha functional connectivity change between brain regions following spinal manipulation (SM). The circular graph plot shows the phase lag index (PLI) of the functional connectivity post-SM. Greater values indicate higher PLI in the circular graph, located to the right in the figure. The visualization of the networks in the cortex shows the functional connectivity between the regions within the brain; red highlighted areas include the default mode network (DMN) with labels to illustrate localization within brain regions. The red dots in the cortex visualization represent the brain regions, which show significant results with bold labels, while the lines connecting them represent the functional connectivity from the PLI metrics, between brain regions. Left medial orbitofrontal (L MOF), right medial orbitofrontal (R MOF), left lateral orbitofrontal (L LOF), right lateral orbitofrontal (R LOF), left parahippocampal (L ParaH), right parahippocampal (R ParaH), left isthmus cingulate cortex (L ICC), right isthmus cingulate cortex (R ICC), left precuneus (L Precun), right precuneus (R Precun), left posterior cingulate Cortex (L PCC), right posterior cingulate cortex (R PCC), left rostral anterior cingulate cortex (L RACC), and right rostral anterior cingulate cortex (R RACC).

Figure 5 illustrates the brain regions whose functional connectivity from the PLI metric was changed from pre to post for the SM session (*t* = 10.45, *p* = 0.005). In order to get a better understanding of the changes found, the results were visualized at the cortex level with the connected brain regions using EEGNET version 1 [64] and circular Graph for the circular graph plots [66].

Changes in PLI functional connectivity exhibited increases after SM in the alpha band between ‘left parahippocampal - left posterior cingulate cortex’, ‘left parahippocampal - right posterior cingulate cortex’, ‘right parahippocampal - left posterior cingulate cortex’, ‘right parahippocampal - right posterior cingulate cortex’. Table 4 shows the effect size (Cohen’s d) occurring from these changes in functional connectivity.

## 4. Discussion

This study investigated the acute effects of SM on the functional connectivity of various brain regions in subacute to chronic stroke patients. Based on the authors best knowledge, this is the first study to investigate the neural changes via functional connectivity using resting-state EEG data in subacute to chronic stroke patients following SM.

### 4.1. Increased Functional Connectivity between Brain Regions within the DMN

Gay et al. 2014 have previously used fMRI to investigate the effect of manual therapies, including SM, on functional connectivity in healthy subjects. They have shown immediate changes in functional connectivity between certain brain regions, including regions of the DMN. For instance, increased connectivity between the left posterior cingulate cortex and left anterior insula, where the posterior cingulate cortex is part of the DMN. These findings occurred alongside decreased pain perception. Studies investigating the function of the DMN in stroke patients have mainly found decreased functional connectivity, compared to healthy subjects, in various brain regions and disruptions during rest within this network is linked with neurologic diseases like stroke [18,21,23,24,25]. Stroke has often been shown to lead to chronic pain and SM has been studied in relation to relieving pain [67]. This pain alleviation after SM is often believed to be related to increased pain threshold [23,68,69]. The current results may now suggest that the observed increased pain threshold in stroke patients reported by previous studies may have been due to the increased functional connectivity within the DMN [23,25]. The fact that the changes happened in the alpha band is expected, as this frequency band reflects brain activity during a relaxed state. This was also where the dominant frequency content existed, seen from the analyzed frequency bands, looking at the PSD analysis in Figure 4 [27,33].

Specific brain regions, such as the posterior cingulate cortex and parahippocampal, whose connectivity was shown to increase after SM, are believed to be involved in pain modulation and the development of chronic pain syndrome via functions that are implicated in chronic pain, including introspection, emotion, and memory [25,70]. Altered dynamics of the DMN is therefore believed to be a part of chronic pain development and can be seen as the maladaptive neuroplasticity of the adult brain caused by a disease, showing as a loss in functional connectivity [70,71]. The lower DMN connectivity found in the Ctrl session may be explained by the fact that the brain of chronic pain patients is never fully at the resting state [72], as the pain perception is involved in the background activity of the brain during rest in these patients. Conversely, in healthy subjects, regions of the DMN shows higher connectivity during rest, as this network is believed to reflect mind attention, memory, and self-processing [25,72]. During rest, a counter intuitive emerging stimulus, not related to the normal function of the DMN at rest, may perturb the resting state of the brain, as the brain may start processing the information conveyed by the stimuli. This is likely because the perception of pain is not being coupled with an exogenous noxious stimulus to background functions at rest [70].

As it is unknown whether the subjects in this study were in pain at the time of the study, nor whether this changed after SM, other explanations of the results of this study should be considered. For example, abnormal connectivity in the posterior cingulate cortex and hippocampus has been identified in early Alzheimer’s disease and mild cognitive impairment patients. Reductions in functional connectivity from both the posterior cingulate cortex and hippocampus to the other regions in the brain as well as between these two regions are thought to be among primary factors in episodic memory impairment associated with early Alzheimer’s disease [73]. It is therefore possible that the increased functional connectivity found after spinal manipulation in the current study may reflect improved episodic memory for the stroke patients. In addition, both imaging and animal experiments link especially the posterior cingulate cortex and hippocampus parts of the DMN to spatial representation and navigation, which might have been altered following SM [21]. Future studies are needed in order to investigate whether SM leads to altered chronic pain perception, cognitive improvements, and/or improved spatial navigation in this population.

### 4.2. Limitations and Future Work

Limitations in the study design and analysis of the results of this study should be acknowledged. As the analyzed data are from resting-state EEG signals, it can only provide information about the underlying resting-state organization of functional connectivity between brain regions. It should also be kept in mind that resting-state data are more abstract to interpret, as there is no specific goal for the subject or hypothesis to be investigated. A direct approach would be to investigate specific, task-related experiments, which would examine whether the subjects could improve in a specific task or give information about pain states. This would also give a clearer picture of the effects and implications of SM and its clinical use in rehabilitation of stroke patients. However, this study sheds light on the potential neural mechanisms, which may be affected by SM in the short-term [74].

Estimation of functional connectivity within the sensor level suffers from smearing effects of volume conduction, as mentioned in Section 2.4.2, but even in the source level from solving the inverse problem, trivial spatial correlations still exist, decreasing with distance and affected by the sampling density [75,76]. For this reason, it is recommended to use connectivity measures that account for field spread and volume conduction [76]. PLI is one of these measures and it has the properties of an undirected connectivity metric [77]. Directed measures, like Granger causality or transfer entropy, could provide further insight into describing the architecture of the functional connectivity [77]. However, such methods have been shown to be quite sensitive to noise and variation in recording gains across EEG sensors [78]. This may also explain the non-significant results in the present study from the coherence analysis, as coherence is affected more by montage effects and volume conduction, which are not robust to the effects of volume conduction [29,79].

Even though a generalized head model for the entire subject pool has been used in various studies and known to be a common method for EEG source level analysis, forward modeling of the head and dipole localization does not take the anatomical differences across subjects into account. This source of error is less dramatic when a high sampling density in EEG recording has been used [53,75].

To have a representative group of participants, we included a very broad range of subacute to chronic stoke survivors with a wide range of time since their stroke and different lesion sites within right or left hemisphere. However, the effect of SM may be different in patients with early stages of stroke, as neuroplasticity is more pronounced in the first three months after onset of the stroke [80,81,82,83]. Due to the wide heterogeneity of the subject pool in this initial study (cf. Table 1), we did not focus on between subject factors, which may have been difficult to control. Therefore, factors such as laterality of the stroke and time of the event have not been studied. Investigation of inter-individual differences in response to SM is a very relevant area of research, provided that the study has the required statistical power and is well-controlled for potential confounding factors. As the aim of this study was to investigate the effects of a single session of SM in stroke patients, healthy subjects were not included in the study design. Another control group consisting of healthy subjects might have proven beneficial in gaining insight to see if the same effects would be seen, but this would also widen the scope of the study, as other effects might be induced within a healthy population. Likewise, the absence of subjective pain scores on, for example, pain and cognitive states in this study did not allow us to substantiate our interpretation of the results.

## 5. Conclusions

The aim of this study was to explore the effects of chiropractic SM on resting-state EEG functional brain connectivity in subacute and chronic stroke patients. From the study, EEG has been shown to be a potential method for monitoring neural changes following SM using the non-linear coupling functional connectivity metric PLI. Furthermore, an altered resting-state functional connectivity following a single session of chiropractic SM was found in subacute to chronic stroke patients, and the observed changes were significant within the DMN. The results showed an increased functional connectivity between the posterior cingulate cortex and parahippocampal areas, which has previously been found to be an important link within the DMN in relation to chronic pain modulation, episodic memory consolidation and/or spatial representation, and navigation. This increased functional connectivity may therefore be ascribed to SM-altering chronic pain, processing episodic memory consolidation and/or spatial representation and navigation. Although clear neuroplastic changes in stroke patients occur following chiropractic care observed from EEG functional connectivity, future research should elucidate the underlying mechanisms and clinical significance of the observed changes.

## Figures and Tables

**Table 1:** Characteristics for each patient participating in the study. Middle cerebral artery (MCA) and anterior cerebral artery (ACA).

Subject Number	Age (Years)	Type of Stroke	Area Involved	Affected Hemisphere	Time Since Event (Months)
1	60	Ischemic	MCA	Right	24
2	41	Ischemic	MCA	Left	19
3	62	Hemorrhagic	MCA	Left	7
4	56	Ischemic	MCA	Left	4
5	34	Ischemic	MCA	Left	42
6	45	Hemorrhagic	MCA	Right	23
7	51	Ischemic	ACA	Left	25
8	60	Ischemic	MCA	Left	12
9	59	Ischemic	MCA	Left	43
10	58	Ischemic	MCA	Right	6
11	54	Hemorrhagic	MCA	Right	24
12	51	Ischemic	ACA	Right	18
13	46	Ischemic	MCA	Right	4
14	68	Hemorrhagic	MCA	Right	60
15	75	Ischemic	ACA	Left	12
16	36	Ischemic	MCA	Right	18
17	48	Hemorrhagic	MCA	Left	24
18	31	Ischemic	MCA	Left	22
19	61	Hemorrhagic	MCA	Right	5
20	64	Ischemic	MCA	Left	7
21	33	Hemorrhagic	MCA	Right	5
22	49	Ischemic	MCA	Right	3
23	48	Hemorrhagic	MCA	Right	24
24	56	Hemorrhagic	MCA	Right	5

**Table 2:** Default mode network brain regions and abbreviations.

Brain Region	Abbreviation
Left Medial Orbitofrontal	L MOF
Right Medial Orbitofrontal	R MOF
Left Lateral Orbitofrontal	L LOF
Right Lateral Orbitofrontal	R LOF
Left Parahippocampal	L ParaH
Right Parahippocampal	R ParaH
Left Isthmus Cingulate Cortex	L ICC
Right Isthmus Cingulate Cortex	R ICC
Left Precuneus	L Precun
Right Precuneus	R Precun
Left Posterior Cingulate Cortex	L PCC
Right Posterior Cingulate Cortex	R PCC
Left Rostral Anterior Cingulate Cortex	L RACC
Right Rostral Anterior Cingulate Cortex	R RACC

**Table 3:** The results of the non-parametric cluster-based permutation test comparing functional connectivity within the default mode network (DMN) for phase lag index (PLI) and coherence. The functional connectivity was compared from pre- to post-intervention (spinal manipulations (SM) and control (Ctrl)). The extent of changes from pre to post. The analysis was performed within alpha, beta, and gamma frequency bands. Positive (+clusters) and negative (−clusters) cluster count are shown, with *t*-value and *p*-values. Significant results (*p* < 0.05) were highlighted with bold text. Ctrl denoted the comparison from pre to post in the Ctrl session, while SM denoted the comparison from pre to post in the SM session.

*p* < 0.05		+Clusters	*t*-Value	*p*-Value	−Clusters	*t*-Value	*p*-Value
**PLI**							
Alpha	Ctrl	0	-	-	3	−3.42	0.474
	SM	4	10.45	0.005	3	−2.37	1.000
Beta	Ctrl	2	3.35	0.450	0	-	-
	SM	2	2.38	1.000	3	−3.21	0.490
Gamma	Ctrl	3	2.45	1.000	4	−3.36	0.518
	SM	1	2.15	1.000	2	−2.45	1.000
**Coherence**							
Alpha	Ctrl	2	2.48	0.542	0	-	-
	SM	1	2.14	0.932	0	-	-
Beta	Ctrl	0	-	-	0	-	-
	SM	0	-	-	1	−2.52	0.399
Gamma	Ctrl	2	2.53	0.563	0	-	-
	SM	0	-	-	1	−2.48	0.484

**Table 4:** Effect size (Cohen’s d) for significant clusters within the alpha band for the SM session. Left parahippocampal (L ParaH), right parahippocampal (R ParaH), left posterior cingulate cortex (L PCC), and right posterior cingulate cortex (R PCC).

Nodes	Pre	Post	Effect size (Cohen’s d)
L ParaH–L PCC	0.0081 (+0.0399)	0.0834 (+0.1275)	0.239
L ParaH–R PCC	0	0.0755 (+0.1260)	-
R ParaH–L PCC	0.0084 (+0.0412)	0.0528 (+0.0943)	0.1359
R ParaH–R PCC	0.0083 (+0.0407)	0.0566 (+0.1013)	0.1481
